# Olfactory dysfunction revisited: a reappraisal of work-related olfactory dysfunction caused by chemicals

**DOI:** 10.1186/s12995-018-0209-6

**Published:** 2018-09-04

**Authors:** Sabine Werner, Eberhard Nies

**Affiliations:** Institute for Occupational Safety and Health of the German Social Accident Insurance, Unit Toxicology of Industrial Chemicals, Alte Heerstrasse 111, 53757 Sankt Augustin, Germany

**Keywords:** Chemically induced anosmia, Formaldehyde, Metals, Occupational exposure, Olfaction disorders

## Abstract

Occupational exposure to numerous individual chemicals has been associated with olfactory dysfunction, mainly in individual case descriptions. Comprehensive epidemiological investigations into the olfactotoxic effect of working substances show that the human sense of smell may be impaired by exposure to metal compounds involving cadmium, chromium and nickel, and to formaldehyde. This conclusion is supported by the results of animal experiments. The level of evidence for a relationship between olfactory dysfunction and workplace exposure to other substances is relatively weak.

## Background

Many occupational groups are reliant upon intact olfactory function in order to perform their work and for their safety. Examples are chefs, gas fitters, firefighters, perfumers, sommeliers, coffee and tea tasters, grocers, workers in the chemical industry, and domestic helpers. The importance of the olfactory function for early detection of hazardous substances with an odour is illustrated by the specific case of an anosmic who lit a cigarette whilst in close proximity to a leaking petrol pipe, thereby causing an explosion [1]. Muttray et al. [2] report the case of a patient who did not become aware of his olfactory dysfunction until his colleagues fled their workplace, to him for no apparent reason, owing to an intense solvent smell. In Germany, an assessment of olfactory function is a requirement for persons applying for certification of their fitness to perform fumigation [3], and loss of olfactory function constitutes grounds for example for the discharge of members of the US military, including reservists, and of coastguard employees [4].

## Diagnosis and assessment of olfactory dysfunction

Assessment of olfactory function and diagnosis of olfactory dysfunction requires, firstly, a detailed medical history and examination by an otolaryngologist [5, 6]. The medical history should include information on the triggering events, development, complementary symptoms, surgical operations, medication and toxicants. The ENT diagnosis comprises medical status, endoscopy of nose and nasopharyngeal space and evaluation of the olfactory cleft. If a neurological disorder is suspected, an examination by a neurologist including tests of cognition and memory could be necessary. Secondly, a validated test method is needed that enables subjective sensory perceptions to be quantified objectively. This is essential for a standardised distinction between normosmia (normal olfactory function), hyposmia and anosmia (impaired olfactory function and its complete loss, respectively), and hyperosmia (olfactory oversensitivity). A screening of global taste function (retronasal smelling) is also advantageous owing to the close connection between smell and taste (patients complaining of a dysfunctional sense of taste are in fact often suffering from olfactory impairment).

Only in the last three decades have standardised and practicable psychophysical tests for humans been developed. Of these, UPSIT (University of Pennsylvania Smell Identification Test [7]) and the “Sniffin’ Sticks” test widely used in Europe [8, 9], are important examples.

“Sniffin’ Sticks” are felt sticks that release aromatic substances when the cap of the stick is removed. In conventional form, they permit threshold, discrimination and identification tests; the last two of these are above-threshold tests. The threshold test indicates the concentration above which an odour is sensed (the sensory threshold). As standard, n-butanol or phenyl ethyl alcohol (rose scent) are used for testing. The non-verbal discrimination test examines the ability to distinguish between odours. In the identification test, 16 odours are tested for recognition [10]. This is a structured, reliable and validated test system that is widely used in Europe and is readily available. Extensive validation studies and defined standard values exist for this test [11, 12]. Regularly updated standardised values are published for example on the website of the Interdisciplinary Center Smell & Taste (University Clinic Dresden) [13].

A similar identification test used in America is the UPSIT method, in which up to 40 odorants, microencapsulated on a sheet of paper, are released by scratching with the point of a pencil. In this test, the various odorants must be identified with reference to a list of four terms per substance. The test kit has a long shelf life, is very well validated and is widely used. The UPSIT test does not require clinician supervision and is therefore very convenient. International versions are also available; they have however rarely been validated specifically for individual countries. A drawback of the test is that it studies only the identification of odours.

Where patients might not be able to comply with psychophysical testing, or in medico-legal assessments, the olfactory dysfunction can be assessed objectively by recording electrical activity of the brain (olfactory event-related potentials, OERPs) following presentation of odours. This method requires virtually no active participation on the part of the test subject, and has been used since the 1970s. It involves the application of olfactory stimuli to the nose of the test subject with the aid of an olfactometer. The stimuli trigger corresponding OERPs, which can then be registered on electrodes applied to the test subject’s head. The use of an electro-olfactograph (the recording of generator potential via an electrode in contact with the olfactory epithelium) is limited to research. Olfactory functional imaging methods such as PET (positron emission tomography) and fMRI (functional magnetic resonance imaging) are also largely limited to research applications [5, 6].

Important information on the toxic properties of inhaled substances that may affect olfaction has been obtained from experiments on animals. Besides histological analyses of the olfactory epithelium, the results of behavioural tests are also relevant. Since the laboratory animal is not able to communicate actively to the researcher whether or not it senses an odour, an operant conditioning test is usually performed. In such a test, mice for example are first taught to expect a reward (such as water following restricted access to water) after sensing a certain olfactory stimulus. The animals are then presented with other odours that are not followed by a reward, in addition to the odour that they have learnt to associate with the reward. Where the animal has recognised the correct odour and looks for its reward, this can be registered, for example by means of a light barrier (for a detailed description, see Kuner and Schaefer 2011 [14]; an up-to-date overview with detailed test protocols can be found in Zou et al. 2016 [15]).

## Work-related olfactory dysfunction

The prevalence of olfactory dysfunction in the wider population is estimated at around 5% (for functional anosmia) [16]. It is considerably more common among older people, and around a quarter of the population aged over 50 exhibit an impaired sense of smell. The results of recent studies suggest that specific anosmias, the failure to sense a specific odour, are far more prevalent than was previously assumed and are the norm rather than the exception [16, 17]. The proportion of olfactory dysfunction caused by occupational exposure remains unclear. Figures for olfactory dysfunction caused by exposure to the effects of chemicals and pharmaceuticals fluctuate between 0.5 and 5% of all cases [18]. According to a large-scale survey of all ear, nose and throat clinics in the German-speaking world, covering 79,000 patients, 2% of the cases had toxic causes [19]. A recent Belgian publication covering a substantially smaller collective of 496 patients with exclusively non-sinusoidal complaints from a specialist clinical centre assumed that 3.4% were toxic in origin [20]. “Idiopathic” cases of olfactory dysfunction, which are put at between 10 and 25%, may however include cases of chemically induced damage caused by workplace exposure not classified as such [21, 22]. In his paper on functional testing and dysfunction of olfaction, Herberhold [23] assumed several decades ago that pathologically elevated olfactory thresholds were present in around 30% of workers in the metals and chemical industry, possibly rising to around 50% with increasing age and increasing duration of exposure to the hazardous substances. Today’s working conditions and exposures are clearly not comparable with those of the 1970s; in her review however, Dalton 2010 [24] also cites a questionnaire and survey conducted in 1995 among 712,000 individuals in Canada and the USA which revealed that factory workers of all ages reported a weaker sense of smell and performed significantly worse in an olfactory test than members of other occupational groups.

According to Herberhold [23], thermal, mechanical and chemical noxae may lead to olfactory dysfunction, the effects triggered by chemicals being more pronounced the more active the chemical substance, the smaller the particles, and the longer the duration of exposure of the sensory apparatus to them. Occupational exposure to numerous industrial chemicals, notably those that are irritative and corrosive to the mucous membranes or harmful to the nerves, is associated with the incidence of olfactory dysfunction (see for example Klimek et al. 1999 [25] and Muttray et al. 2006 [26]). In his overview, Amoore [27] lists over 100 substances presumed capable of causing olfactory dysfunction. This information is based almost entirely upon case reports rather than on large-scale studies. The discussion below therefore gives consideration to the substances associated with occupational olfactory dysfunction that have been studied under standardised conditions in epidemiological studies and studies of test subjects. The results of relevant animal experiments concerning the substances identified in this way that to some extent permit conclusions regarding the possible mechanism of action are also presented.

## Industrial chemicals with a potential impact upon olfaction

In order to identify industrial chemicals exposure to which may potentially lead to olfactory dysfunction, a literature survey was conducted in the Pubmed database in order to identify substances characterised as olfactotoxic primarily on the basis of epidemiological studies and studies of test subjects conducted under standardised conditions. The terms “anosmia”, “hyposmia”, “dysosmia”, “smell disorders”, “olfactory function”, “olfactory dysfunction” and “olfaction disorders” were each combined in the search with “occupational”, “professional” and “workplace”. In the second step, the substances identified in this way were used as search terms in combination with the relevant clinical pictures (see above) in order to identify animal experiments. Relevant studies in the bibliographies of the identified literature were considered. Pharmacological and environmental studies associated with the identified industrial chemicals were also included. Case reports, for example concerning accident-type events involving very high exposures, were disregarded.

A comprehensive overview of long-term effects of occupational exposures to metals and olfactory toxicity can be found in the reviews by Gobba 2006 and Sunderman 2001 [21, 28]. One aspect addressed by the recent review in Doty 2015 is likewise the influence of exposure to neurotoxic substances in the environment or at the workplace upon the sense of smell [4]. Accordingly, the studies referred to in these publications will not be considered in further detail in the present paper. The focus here lies on the epidemiological and animal studies not stated there. The latter will be described in detail. Human studies on olfactotoxic effects caused by chemicals are summarised in Table 1.

**Table 1:** Work-related olfactory dysfunction: Human studies (in chronological order)

Human studies Reference	Occupation	Exposure (mg/m^3^)	Exposed workers, residents, patients	Controls	Test method	Results
Cadmium/nickel						
^a^Friberg 1950 [29]	Battery factory	3–15 Cd 10–150 Ni	43 (group 1: longer employment 9–34 years) 15 (group 2: shorter employment 1–4 years)	–	Test with odour samples (coffee, perfume, peppermint and petrol)	Group 1: 37% impaired sense of smell (32.6%: anosmia). Group 2: 6.7% (one case) partially impaired sense of smell.
^a^Baader 1951 [30])	Battery factory	N. i.	8	–	Test with odour samples (no further details)	4 hyposmic or anosmic.
^a^Potts 1965 [31]	Battery factory	Cd dust and fumes: 0.6–236 (1949) < 0.5 (1950) < 0.1 (1956)	70	–	N. i.	64% anosmic.
^a^Liu et al. 1985 [32]	Cadmium smelters	Cd oxide: 0.004–0.187	65	–	N. i.	21.5% anosmic.
^a^Adams and Crabtree 1961 [33]	Battery factory	Cd: 0.028–2.76 Ni: 0.0016–0.056	106	84	Threshold test with phenol	27.3% anosmic (Co: 4.8% anosmic)
^a^Rose et al. 1992 [34]	Factory producing refrigerating coils	Cd fumes up to 0.3	55	16	Threshold test with n-butanol. Identification test with 10 different olfactory stimuli	13% severe hyposmia (Co: 0%), 44% weakly hyposmic (Co: 31%). Identification test: no significant difference.
^a^Rydzewski et al. 1998 [35] ^a^Sulkowski et al. 2000 [36]	Battery factory	Atmospheric cadmium concentrations: 0.05–2.1	73	43 forestry workers	Threshold identification test with modified Elsberg-Levys method	Significantly impaired ability to detect and identify odours. Overall result: 26% hyposmia, 17.8% parosmia, 1.4% anosmia (Co: 30.2% parosmia, 69.8 normosmia).
^a^Mascagni et al. 2003 [37]	Workers in a cadmium foundry and sintering plant	Cadmium concentrations (max.): 1.530 (1975) -0.0171 (1995); by 1978, the measurement results had already dropped substantially below 1 (at 0.207)	33	39 drivers and storekeepers, 23 welders	Threshold test with phenyl ethyl alcohol Identification test with Wright’s confusion matrices	Olfactory threshold significantly higher in Cd workers than in controls. Identification test: no significant difference (a non-significant impairment in cadmium workers). Overall results: 3.1% anosmic, 3.1% severely hyposmic, 24.1% mildly/moderately hyposmic (welders: 0% anosmic, 4.3% severely hyposmic, 8.7% mildly/moderately hyposmic; Co: 0% anosmic, 0% severely hyposmic, 7.7% mildly/moderately hyposmic).
^a^2 studies from USSR cited in Sunderman [28]: (Tatarskaya 1960) (Kucharin 1970)	Electrolytic Ni refinery Electrolytic Ni refinery	N. i. N. i.	N. i. 458	N. i. N. i.	N. i. N. i.	Frequent olfactory impairment, atrophic nasal mucosa, nasal septal ulceration and sinusitis. Anosmia in 114/251 workers (46%) with chronic sinusitis; less severe loss of smell in other Ni-exposed workers.
Chromium						
Seeber et al. 1976 [53]	Chrome paint plant (manufacture of basic zinc chromate (zinc yellow))	Values exceeded 0.1 CrO_3_ air, and rose at certain points to up to 20 CrO_3_. More precise values are not stated.	5 chronically exposed workers, 14 intermittently exposed workers (not longer than 2 h per day), 5 mask-wearing workers (wearing fine-dust filter masks for the full duration)	9 office workers at the same company, 23 employees at hospital	Test with odour strips (6 different stimuli in 8 concentrations)	Olfactory sensitivity of control group and non-exposed individuals substantially higher than that of exposed individuals. “Significant” relationship between chromium exposure and loss of olfaction. Chronically exposed individuals: damage to the nasal mucous membrane. Intermittently exposed group and workers with a mask: damage to the nasal mucous membrane only in a part.
Seeber and Fikentscher 1980 [54]	See above	Occupational exposure “substantially reduced” by suitable measures (not specified)	3 chronically exposed workers, 9 intermittently exposed workers (not longer than 2 h per day) 4 mask-wearing workers (wearing fine-dust filter masks for the full duration)	7 office workers at the same company, 23 employees at hospital	See above	Confirmation of the relationship between the pathological nasal mucous membrane findings and olfactory dysfunction in 16 workers exposed to different levels. No improvement in the mucous membrane or olfaction.
^a^Watanabe and Fukuchi (1981) [55]	Chromate production plant	Air Cr concentration of 20.17 according to [56]	33	–	T&T olfactometer (odour detection threshold and odour recognition)	“Middle and high grade decrease of odor recognition faculty” in 18 workers (54.5%) including 2 anosmiac, one of whom also complained of a taste disorder. 51% exhibited nasal septum perforations. Relation between degree of olfaction loss and duration of employment in the chromate producing factory.
^a^Kitamura et al. 2003 [56]	Cr plating factory	Average atmospheric concentration was 0.0228	27	34	T&T olfactometer (odour detection threshold and odour recognition) Olfactory perception threshold test	No significant differences for sensory and perception threshold. Significantly higher values for the odour recognition test than those for controls, positive correlation with duration of exposure. None of Cr workers showed nasal septum perforation or ulceration.
Aiyer et al. 2003	Chromium plating industry	N. i.	28	–	N. i.	11 workers anosmic (nasal septal perforation of different magnitudes on all exposed workers, majority with initial symptom of nasal irritation).
Manganese						
^a^Lucchini et al. 1997 [58]	Ferroalloy production plant	Manganese dust exposure: 0.026–0.750 (geometric mean: 0.193)	35	37	Olfactory perception threshold to PM-carbinol (3-methyl-1-phenylpentan-3-ol dilution series)	No significant differences (although it was negatively associated with Mn levels in urine in the exposed group)
^a^Mergler et al. 1994 [59]	Ferromanganese and silicomanganese alloy plant	Manganese dust exposure: 0.014–11.48 Total Mn levels in dust: 0.89 (geometric mean) Manganese content of respirable dust fraction: 0.001 and 1.273 (geometric mean: 0.04)	74 (matched pairs)	74	Olfactory perception threshold to PM-carbinol	Significantly increased olfactory perception among workers compared to controls.
^a^Antunes et al. 2007 [60] ^a^Bowler et al. 2007 [61]	San Francisco/Oakland Bay Bridge welders	Atmospheric manganese levels lay between 0.11 and 0.46 (55% > 0.20)	43 welders	43 (matched by age, sex, education and smoking status from the database of the University of Pennsylvania Smell and Taste Center)	UPSIT identification test	Significantly weaker olfactory function of welders than in controls. Score of 88% of the workers below their individually matched controls. 3% of the welders were anosmic. Better olfactory function in workers with the highest Mn blood levels than those with the lowest Mn blood levels.
Bowler et al. 2011 [62]	Follow-up study three and a half years later	–	26 welders from the San Francisco/Oakland Bay Bridge welder study (13 study participants were no longer working as welders)	–	UPSIT identification test	No significant differences from earlier findings. The blood-manganese levels of the former workers were significantly lower than those of their colleagues.
Sen et al. 2011 [63]	Welders	Cumulative Mn exposure (mg/m^3^x years): Welders 0.881 (± 0.567) Controls 0.002 (± 0.0003)	7 welders	7	MRI	Increased manganese deposition in the olfactory bulb and in other regions of the brain. No statistically significant differences in the olfactory test between the two groups (data not shown).
Guarneros et al. (2013)	Persons living in proximity to a manganese plant	Elevated manganese hair concentration (mean 0.00973 vs. 0.00101)	30 persons living within a one-kilometre radius of a Mexican manganese mine	30 controls living more than 50 km away	Sniffin’ Sticks olfactory test series (threshold, discrimination and identification tests)	Significantly diminished olfactory function in those living close to the manganese mine (in threshold, discrimination and identification tests).
Lucchini et al. 2012 [65]	Residents of Valcamonica: Italian region, marked by ferrous alloy plants until 2001	Average Mn atmospheric and soil values at the time of the study: 0.0495, 958	154 young people aged between 11 and 14	157 young people from Lake Garda region	Sniffin’ Sticks (identification test)	Significantly poorer olfactory function associated with Mn content in the soil.
Iannilli et al. 2016	Valcamonica (see above) and Bognolo Mella: regions with a history of high Mn contamination	–	9 young people from Valcamonica and Bagnolo Mella exposed to manganese	4 young people from the Lake Garda region	Sniffin’ Sticks (identification test), fMRT (response activity to olfactory tasks)	No significantly different results of the identification test between the two groups. Reduction in activity in relevant olfactory brain regions. In comparison with a larger control group from a database, significant differences were observed with regard to the size of the olfactory bulb and in the olfactory test involving Sniffin’ Sticks.
Casjen et al. 2017 [67]	Blue collar workers	Median: 58.3 μg/m^3^ x years (interquartile range 19–185 μg/m^3^ x years)	354	1031	Sniffin’ Sticks (identification test)	No relevant association of former Mn exposure at relatively low levels with impaired olfaction.
Zinc						
Pyatayev et al. 1971 [73]	Zinc production plant	Zinc oxide, zinc sulfate and metal dusts. Co-exposure to further strongly corrosive and irritant substances	301	63	Olfactometer employing mint and dilute acetic acid	Significant elevations of sensory thresholds. Elevations were highest among workers responsible for roasting the zinc ore.
Anonymous 1938, ^a^Tisdall et al. 1938 [74, 75]	(Treatment in special clinics with zinc in nasal spray for prevention of poliomyelitis infection)	Nasal spray solution containing 1% zinc sulfate and 0.5% tetracaine	5233 children (4713 (received two sprayings) + 520 (received one spraying))	6300	N. i.	No more than a quarter exhibited temporary anosmia.
^a^Tisdall et al. 1938 [75]	(Patients treated by the same otolaryngologists, but in their private practices)	See above	5000 children and adults	–	N.i.	6 months following treatment: 44 of the patients were permanently anosmic (52 with disturbances of smell and taste).
Davidson et al. 2010 [76]	(Nasal Dysfunction Clinic of the University of California in San Diego)	Intranasal zinc gluconate gel	10 patients	–	n-Butanol threshold UPSIT (identification test)	3 subjects with anosmia and 7 with hyposmia.
Alexander et al. 2006 [77]	(See above)	Intranasal zinc gluconate gel	17 patients	–	n-Butanol threshold, identification test employing seven known odorants and one substance for testing the trigeminal function, UPSIT (9 patients)	Impaired olfaction in all patients: 7 patients anosmic, 10 hyposmic. Trauma and infection were ruled out as causes of hyposmia and anosmia diagnosed in 15 of these patients in the relevant tests.
Jafek et al. 2004 [78]	(Special “Taste and Smell Center” at the University of Colorado School of Medicine)	Use of zinc gluconate gel	10 patients	–	N. i.	Suffering of severe hyposmia in conjunction with parosmia or anosmia following use of zinc gluconate gel.
“Pesticides”						
^a^Calvert et al. 1998 [102]	Structural fumigation workers	Lifetime duration of methyl bromide and sulfuryl fluoride exposure: 1.2 and 2.85 years Sulfuryl fluoride values of earlier NIOSH measurements: below 20	123	120	UPSIT (identification test)	Significantly weaker olfactory function in workers with high sulfuryl fluoride exposure over the year preceding examination.
Quandt et al. 2016 [104]	Latino farmworkers	Lifetime exposure. Total years in jobs involving pesticide exposure: farmworkers 13.11 (mean), non-farmworkers 3.84 (mean)	304	247	Sniffin’ Sticks Kit (identification test and threshold test with n-butanol)	No difference in odour identification performance but significantly higher odour thresholds.
Formaldehyde						
^a^Holmström and Wilhelmsson 1988 [111]	Workers at a chemical plant where formaldehyde and products based on formaldehyde were produced	0.05–0.5 formaldehyde (for the group of workers impregnating paper up to 1)	70	36 (office workers with 0.9 mean exposure to formaldehyde)	Sensory threshold test employing pyridine	Significantly reduced olfactory function.
^a^Holmström and Wilhelmsson 1988 [111]	Work with glued wood in the production of furniture	0.2–0.3 (formaldehyde) 1–2 (wood dust)	100	36	See above	Significantly reduced olfactory function (but no difference between the formaldehyde and the formaldehyde-wood dust groups).
^a^Hisamitsu et al. 2011 [112]	Medical students during cadaver dissection	0.64–1.2 (middle of the laboratory), 0.28–0.88 (in the corners)	41	–	Nagashima jet nebulising olfaction test with bromine (detection threshold)	Significantly diminished olfactory function (32%, temporary).
Kilburn et al. 1985 [113]	Histology technicians	0.25–2.38 (exposure to other solvents such as xylene, toluene and also chloroform)	76	56	Questionnaire	Significantly more self-reported frequent reduced sense of smell in histology technicians (32% of the women who were exposed to formaldehyde for 1 to 3 h and likewise 32% of the women who were exposed for over 4 h stated that their olfactory function was diminished, 5% Co).
Edling et al. 1988 [114]	Workers at different plants (laminate plants, particle board plants)	0.1–1.1 with exposure peaks of up to 5 (formaldehyde), 0.6–1.1 (wood dust)	75	25	Histological examination	Pathological changes of the nasal mucous membrane in 72 individuals.
Acrylates						
^a^Schwartz et al. 1989 [119]	Workers at a chemical facility manufacturing acrylates and methacrylates	0.0416–232.96 ethyl acrylate and acrylic acid (mostly around 30 for acrylic acid and 20.8 for ethyl acrylate)	55 (high) 164 (low)	512	UPSIT (identification test)	No association in the first instance between exposure and results of olfactory tests.
^a^Schwartz et al. 1989 [119] embedded case-control study	See above	See above	77	77		Dose-effect relationship between olfactory dysfunction and cumulative exposure; the effect appeared to be reversible. Workers who had never smoked had the highest relative risk of olfactory dysfunction.
Muttray et al. 1997 [120]	Chemical workers in acrylic sheet production	Methyl methacrylate (MMA): 104–416 (1988), 41.6–208 (1989–1994), 9.6 ±7.1 years (mean duration of MMA exposure)	175	88	Rhino Identification Test (6 tested aromas, very similar in design to the UPSIT test)	No significant difference between exposed workers and control group.
Muttray et al. 2007 [121]	Healthy volunteers	208 MMA and room air in an exposure chamber at an interval of one week, in each case for 4 h	20	–	Olfactory threshold for n-butanol (Sniffin’ Sticks)	Olfactory threshold: no changes.
Styrene						
^a^Cheng et al. 2004 [123]	Injection-moulding workers exposed to acrylonitrile-butadiene-styrene (mainly manufacture of computer shells)	–	52	72	Olfactory threshold test employing 1-butanol, CCCRC olfactory test (identification)	Slight but significant reduction in olfactory function (threshold) at the end of the shift (whereas the initial situation at the beginning of the shift had been the same). The identification test revealed no differences before and after the shift.
^a^Dalton et al. 2003 [124]	Workers in the reinforced-plastics industry	Mean airborne styrene concentrations: 89.2 (day 1), 106.1 (day 2) Means and ranges of historic exposures to airborne styrene for individual workers (*n* = 52): 57.37 (15.16–134.23) Cumulative mean exposure: 675.48 (59.75–1420.24) Peak year exposure: 112.58 (22.52–331.25)	52	52	Olfactory threshold for phenyl ethyl alcohol and styrene, retronasal odour identification test with five stimuli, identification test with 20 odorants	No significant differences between styrene-exposed workers and matched controls in the results of the phenylethyl alcohol threshold test, retronasal test, or odour identification test. Significantly different odour detection thresholds for styrene among exposed and unexposed groups.
^a^Dalton et al. 2007 [125]	Workers in a reinforced plastics boat-manufacturing facility	Calculated effective mean concentration of styrene in air: 43.3–108.25 (measured airborne styrene concentration for this group: 303.1–346.4)	15	15	Olfactory threshold for phenyl ethyl alcohol (PEA) and styrene, identification test with 18 odorants	No significant difference in olfactory threshold for PEA. Significant difference in the threshold test involving styrene among exposed and unexposed groups. Significant difference in the identification test between workers with high vs. low exposure (possible explanations: more individuals with poor values in both groups than in normal population, cultural and educational differences between the two groups).
Organic solvents and mineral oil products						
^a^Schwartz et al. 1990 [130]	Workers in paint manufacturing facilities	Solvents: toluene, xylene and methyl ethyl ketone Lifetime hydrocarbon dose in ppm years (averages at the two production plants): 180 (±128), 97 (±70)	187	–	UPSIT identification test	Significant dose-dependent deterioration in olfactory function with rising lifetime exposure among workers who had never smoked.
^a^Sandmark et al. 1989 [131]	Painters	No quantitative exposure measurement (described as “low to moderate”)	54	42	UPSIT identification test	No statistically significant impairment of olfactory function after adjustment for age and smoking habits.
Studies cited in Muttray et al. 1998 [133]: (Dragomitretzky et al. 1970) (Kmita 1953) (Latkowski et al. 1981 [134])	Workers at a shoe factory, petroleum chemistry workers	A petroleum mixture (“Galoscha”), ethyl acetate and butyl acetate (solvent concentration: 220–300), rubber adhesive containing petroleum (up to 3000) N. i.	216 205 547	N. i. N. i. 100 (cotton industry workers)	N. i. N. i. Smell and taste tests	31% suffered the loss of olfactory function, and among the remainder, olfactory function was impaired in comparison to a control group. Olfactory dysfunction as a result of rhinitis arose after a few years. Hyposmia in 238 (43.5%) and anosmia in 50 (9.2%) subjects. Dysgeusia in 319 (58.3%). (Co: dysgeusia in 69%, anosmia in 24% of subjects).
^a^Ahlstrom et al. 1986 [132]	Tank cleaners	Mineral oil products (heavy and light oils, hydrocarbon content: 240–1615)	20	40	Threshold test, perceived odour intensity test (pyridine, dimethyl disulfide, n-butanol and heated oil vapour)	Elevated olfactory threshold values for heated oil vapour and n-butanol in comparison to controls (for n-butanol within the normal range, oil vapour had not been studied in this respect before). Higher threshold values for all tested substances exhibited by tank cleaners exposed one day prior to the test in comparison to individuals exposed earlier. Lower odour intensity values at the lowest stimulus concentrations for all 4 substances in comparison to those of control subjects (“odour intensity recruitment”).

*N.i.* not indicated, *Co* control group, ^a^studies which have already been discussed in the reviews by Sunderman, Gobba and Doty [4, 21, 28]

### Cadmium and nickel

A considerable number of epidemiological studies demonstrate an association between exposure to metals in the form of dusts and vapours, and occupational olfactory dysfunction. Anosmia and hyposmia were diagnosed for example among workers exposed to dust containing nickel or cadmium in plants for the production of alkaline batteries, nickel refineries, and the cadmium industry.

#### Human studies

##### Cadmium exposure

Nine epidemiological studies published in the period from 1950 to 2003 [29–37] address the association between workplace cadmium/nickel exposure and olfactory impairment. The older studies lack clear information on the test methods and comparisons with non-exposed subjects [29–32]. Anosmia/hyposmia was detected in a significantly high proportion of the exposed workers in all human studies. Differentiated sensory and identification tests were conducted in three studies [34–37]; Rydzewski et al. and Sulkowski et al. [35, 36] describe the same data, which can therefore also be counted as one. Whereas Rose et al. and Mascagni et al. [34, 37] demonstrated that the values obtained in the sensory threshold test were considerably higher among the workers than in the control group, the identification test revealed no significant differences. The sensory threshold test for n-butanol or phenyl ethyl alcohol is regarded as an instrument for diagnosing the function of the peripheral olfactory receptor neurons. According to this interpretation, the ability to identify odours is based primarily upon the processing of olfactory information in the cerebral cortex. This absolute distinction is problematic however, since the sensory threshold test also encompasses complex functions of the central nervous system, and the identification of odours is dependent upon the activity of the olfactory receptor neurons.

Many of the epidemiological studies that describe the incidence of olfactory dysfunction following exposure to cadmium are older. In the 1950s and 1960s, occupational exposure to cadmium was appreciably higher than it is today, for example 0.6–236 mg/m^3^ in a battery factory [31]. The threshold limit values (TLVs) proposed by the American Conference of Governmental Industrial Hygienists (ACGIH) stated a limit concentration of 50 μg/m^3^ in 1975 and 10 μg/m^3^ as of 1995. Conversely, more recently published measured exposure values of 0.004–0.187 mg/m^3^ [32], 0.3 mg/m^3^ [34] and 1.53 mg/m^3^ (1975) and 0.0171 mg/m^3^ (1995) [37] demonstrate that damage to olfactory function may arise even at low concentrations.

##### Nickel exposure

With the exception of the studies stated by Sunderman, we are not aware of any studies into the association between workplace nickel exposure and olfactory dysfunction. Sunderman [28] cites two studies from the former Soviet Union from 1960 and 1970, according to which workers exposed to nickel in electrolytic nickel refineries exhibited olfactory dysfunction/anosmia in addition to atrophy of the nasal mucous membrane, chronic sinusitis and ulceration of the nasal septum.

In the studies cited concerning cadmium, the workers were often exposed not only to cadmium, but also to nickel (see above). Whereas in the 1940s, occupational nickel exposure of 10–150 mg/m^3^ for example was possible [29], considerably lower values of up to 0.056 mg/m^3^ were measured in the publication by Adams and Crabtree in 1961 [33]. ACGIH currently sets a TLV of 1.5 mg/m^3^ for the inhalable fraction of nickel.

#### Animal experimental studies

##### Cadmium exposure

Following inhalation tests on rats (250 and 500 μg/m^3^ CdO, 5 h per day, 5 days per week for 20 weeks), an elevated cadmium level was determined in the olfactory bulb. This was accompanied neither by significant histopathological changes in the mucous membrane, nor by a reduction in the olfactory function [38].

In another animal experiment, administration of 400 μg of CdCl_2_ to mice by intranasal instillation resulted in partial damage to the olfactory epithelium, reversible loss of olfactory discrimination, and specific cadmium deposition in the olfactory bulb but not in other parts of the central nervous system [39]. Czarnecki et al. [40] also observed anosmia in a behavioural test following intranasal instillation of a cadmium chloride solution in mice. They further demonstrated a dose-dependent reduction in the odour-induced release of neurotransmitters from the olfactory nerve into the olfactory bulb. Moreover, a 20% drop in the dendrite density of the olfactory epithelium was described at the highest dose (20 μg CdCl_2_). In further experiments, Czarnecki et al. demonstrated a clear cadmium accumulation on mice specifically in the olfactory bulb by bilateral instillation of a buffer solution of 20 μg of CdCl_2_ per nostril. The accumulation was still measurable 4 weeks after exposure. A reduction in the axonal terminals of the olfactory receptor neurons was also demonstrated histologically. A decrease in neurotransmitter release in response to olfactory stimulation was detected in vivo on the mice exposed to cadmium (intranasal instillation with 0.2, 2 and 20 μg CdCl_2_) 2, 7 and 28 days after exposure. After the laboratory animals treated with cadmium had exhibited significant olfactory deficits in a behavioural test, these deficits disappeared after two weeks of olfactory training; however, the mice with restored olfaction continued to exhibit damaged projections of the olfactory receptor neurons in the results of optical imaging. Czarnecki et al. conclude from this that restoration of olfactory function is attributable to neuroplasticity: the brain, they assume, has learnt to reinterpret the reduced stimuli appropriately. Such processes of neuronal plasticity could mask severe damage by neurotoxic substances [41].

Cadmium-induced olfactory impairment was also confirmed in fish [42]: after 8-h exposure to 347 ppb of Cd in fresh seawater, coho salmon exhibited not only histological changes to the olfactory epithelium and diminished olfaction in the behavioural test (for example loss of the tonic immobility response to olfactory alarm signals), but also significantly reduced expression of olfactory receptors and increased expression of enzymes involved in the antioxidant reaction in relation to metals. During 48-h exposure to 3.7 ppb, tonic immobility responses were diminished and histological changes to the olfactory epithelium likewise occurred that were not as pronounced as in the highly exposed fish group.

##### Nickel exposure

Inhalation of NiSO_4_ (0.635 mg, 6 h per day, 16 days) caused atrophy of the olfactory epithelium in rats, α-Ni_3_S_2_ additional chronic inflammation of the nasal tissue. Significant impairment of olfaction was not recorded [43–47]. Studies on rats and apes confirm the transport of nickel into the olfactory bulb following inhalation of soluble NiSO_4_ [47]. Following intranasal instillation of ^63^Ni^2+^ in rats, the uptake pathway was tracked from the olfactory epithelium, via the axons of the primary olfactory neurons, into the glomeruli in the olfactory bulb and into further parts of the brain [48]. A maximum ^63^Ni^2+^ transport rate of 0.13 mm/h was measured in the olfactory neurons of pike [49]. In a recent examination by Jia et al. [50], intranasal instillation of nickel sulfate (0.5 and 2.5 mg/kg) in mice led to dose-dependent and time-dependent atrophy of the olfactory epithelium of the turbinate bone, but not of the septum. The sustentacular cells were affected first by apoptotic cell loss on the first day post exposure, the olfactory receptor neurons on the third day; a significant increase in cell proliferation in the olfactory epithelium was detected after 5 to 7 days.

Neuronal signal transduction by calcium and apoptosis is a factor in olfactory impairment by nickel: according to Zhao et al. [51], NiSO_4_ is capable of inducing apoptosis by activation of the death receptor 3 and caspase-8 and subsequent activation of caspase-3; Jia et al. suspect NiSO_4_-induced apoptosis of the olfactory receptor neurons to be attributable to this mechanism. Moreover, according to Gautam et al. [52], Ni^2+^ can reduce the odour-induced calcium influx by inhibition of the T-type Ca^2+^ channels in the olfactory receptor neurons, thereby impairing signal transduction.

### Chromium

#### Human studies

Exposure to chromium is frequently encountered in combination with nickel and other metals. According to Seeber et al. [53], ulcers on the skin and mucous membrane and perforation of the nasal septum caused by chromium were known as long ago as 1826. Few epidemiological studies exist of a possible association between chromium exposure and olfactory dysfunction.

In all 5 human studies known to us, deficits in the olfactory function of the exposed workers were detected that were associated significantly with the chromium exposure and the duration of employment [53–57]. The study by Seeber et al. and the follow-up research by Seeber and Fikentscher of 1976 and 1980 were not mentioned in the reviews by Gobba, Sunderman and Doty, and are described accordingly in more detail here: Seeber et al. (1976) and Seeber and Fikentscher (1980) reported on damage to the nasal mucous membrane and olfactory dysfunction among workers at a chrome paint plant in the German Democratic Republic (GDR) in which basic zinc chromate (zinc yellow) was manufactured [53, 54]. In their comparison between chronically exposed workers (5), intermittently exposed workers (14, not longer than 2 h per day), mask-wearing workers (5, wearing fine-dust filter masks for the full duration), non-exposed individuals (9 office workers at the same company) and a control group (23 employees at a hospital), they determined by means of rhinoscopic examinations that damage to the nasal mucous membrane was evident on all five of the chronically exposed individuals, but occurred only in a part of the intermittently exposed group and among the workers wearing masks, and not at all in the other groups. Olfactory tests involving odour strips for ascertaining the sensitivity to certain substances showed the olfactory sensitivity of the control group and the non-exposed individuals to be substantially higher than that of the exposed individuals. The authors considered this relationship between chromium exposure and loss of olfaction to be “significant”. According to the authors, the dust values measured at the points at which zinc chromate dust was produced clearly exceeded the occupational exposure limit for chromium (VI) applicable in the GDR at this time of 0.1 mg CrO_3_/m^3^ air, and rose at certain points to up to 200 times the occupational exposure limit. More precise values are not stated. Four years later, after violation of the occupational exposure limit in this plant had been “substantially reduced” by suitable measures (not specified), the workforce was examined once again. It was found that in 16 workers exposed to different levels, the relationship between the pathological nasal mucous membrane findings and olfactory dysfunction was confirmed, and that on average, no improvement in the mucous membrane or olfaction was detected [54].

Besides the results obtained by Watanabe and Fukuchi (1981) and Kitamura et al. (2003), in which an impaired olfactory function in workers in the chromate and galvanising industry was detected by means of the T&T olfactometer and which have already been described in detail in Gobba 2006, workers in galvanising are also shown to be affected by olfactory dysfunction in an Indian publication from 2003: the authors reported on 28 workers in the chromium industry aged between 22 and 37 and exposed to chromium for between 5 and 14 years. Of these, all 28 employees exhibited nasal septum perforations, and 11 were anosmic. Information on the level of exposure and on the test method was not provided [57].

Kitamura et al. reported chromium-induced olfactory dysfunction at low workplace concentrations [56]. The exposure values for chromium measured in this study were 0.0047 to 0.059 mg/m^3^. ACGIH set a TLV of 0.05 mg/m^3^ for water-soluble Cr (VI) compounds and 0.01 mg/m^3^ for insoluble Cr (VI) compounds.

#### Animal experimental studies

In his review of the relationship between exposure to metals and nasal toxicity, Sunderman cites an animal experiment on rats. Following 40 days’ inhalation of sodium dichromate (0.2 mg/m^3^, 6 h per day, 40 days), the rats exhibited no morphological nasal changes. Olfaction was not tested [28].

### Manganese

#### Human studies

According to the results of our searches, 10 human studies are available to date that examine the association between manganese exposure and impairment of the sense of smell [58–67]. The study populations encompass not only exposed workers, but also persons living close to a manganese mine and young people living in a region in which manganese emissions of industrial origin were very high prior to 2001. In the two studies conducted on workers in the metals industry [58, 59], in which only the sensory test method was employed, the results were either not significant [58] or, surprisingly, revealed a significantly increased olfaction perception among the workers in measurements of the sensory olfactory threshold [59]. By comparison, use of the olfactory identification test employing Sniffin’ Sticks on welders or the inhabitants of an area with elevated background manganese values revealed significantly poorer values [60–62, 64–66]. In further magnetic or functional resonance imaging studies, elevated manganese deposition in the olfactory bulb was measured on welders [63] and a reduction in activity in relevant olfactory brain regions was measured in young people living in a region exhibiting elevated manganese values [66].

The following recent studies have not yet been discussed in the reviews by Sunderman, Gobba and Doty and will therefore be presented in more detail here:

In a follow-up survey, 26 welders who had participated in the San Francisco/Oakland Bay Bridge welder study [60, 61] were examined three and a half years later by the same methods. Although 13 participants were no longer working as welders, the results obtained in the UPSIT did not differ significantly from the earlier findings. The blood-manganese levels of the workers who were no longer welding were significantly lower than those of their colleagues [62].

For seven welders, increased manganese deposition in the olfactory bulb and in other regions of the brain was demonstrated by means of functional magnetic resonance tomography [63].

Guarneros et al. (2013) conducted a Sniffin’ Sticks olfactory test series encompassing threshold, discrimination and identification tests on persons living in proximity to a manganese plant and exhibiting an elevated manganese concentration (mean 9.73 μg/g vs. 1.01 μg/g) in their hair [64]. Significant differences between the subjects were observed. In this study, 30 persons (non-smokers) living within a one-kilometre radius of a Mexican manganese mine were compared with 30 controls living more than 50 km away. The groups were matched by age, sex and school education; none had previously worked in a job involving manganese exposure. The results of the Sniffin’ Sticks test revealed substantially diminished olfactory function in those living close to the manganese mine.

By means of the identification test employing Sniffin’ Sticks, Lucchini et al. also documented significantly poorer olfactory function associated with the Mn content in the soil on 154 young people aged between 11 and 14 in Valcamonica (Italy). Up until 2001, this region was marked by ferrous alloy plants and the emissions from them (average Mn atmospheric and soil values at the time of the examination: 49.5 ng/m^3^ and 958 ppm respectively). Young people from the region around Lake Garda were tested as the control group [65]. In a further study employing functional magnetic resonance tomography, the activity of the brain in 9 young people from Valcamonica and Bagnolo Mella exposed to manganese was compared with that of 4 young people from the Lake Garda region. In the exposed young people, a reduction in activity in relevant olfactory brain regions was detected, for example in the orbitofrontal cortex and piriform cortex and in further brain regions typically associated with olfactory function, such as the middle frontal gyrus and cerebellum. In comparison with a larger control group from a database, significant differences were also monitored with regard to the size of the olfactory bulb and in the olfactory test involving Sniffin’ Sticks. Reduced activity in comparison with that of the controls was also noted in the regions of the limbic system [66].

In a recent prospective cohort study, Casjens et al. examined the influence of work-related manganese exposure upon the olfactory function. The study population comprised 1385 men, of whom 354 had potentially been exposed to manganese in their earlier occupations. No relevant association was determined between manganese exposure and a deterioration in olfactory function [67].

#### Animal experimental studies

Experiments on pikes and rats showed that following intranasal application of a dilute ^54^MnCl_2_ solution, manganese is absorbed by the olfactory epithelium and transported on into the brain. In the process, manganese accumulates in the olfactory bulb and can be detected after 12 weeks throughout the brain and spinal cord [68–71]. Transport of the manganese from the nasal cavities into the brain requires the axonal projections of the olfactory epithelium’s receptor neurons to be intact [72]. Foster et al. also evaluated the transport of manganese from the olfactory epithelium to the olfactory bulb: a bilateral instillation of 40 μl 200 mM MnCl_2_ in rats leads to an increase in manganese levels in both the olfactory epithelium and the olfactory bulb, and the rats exposed to manganese exhibit decreased performance in the olfactory discrimination task. Manganese accumulation in the olfactory bulb and in other regions of the brain was also demonstrated by MRT studies on non-human primates exposed to aerosolised MnSO_4_ (≥ 0.06 mg Mn/m^3^) [71].

### Zinc

Exposure to zinc in the form of fumes and dust frequently occurs during the manufacture and processing of metals. With the exception of one study published in 1971 in Russian [73], we are not aware of any studies of a possible relationship between the incidence of olfactory dysfunction and occupational zinc exposure. Studies do exist of the frequent incidence of anosmia following medical intranasal application of sprays or gels containing zinc, as do studies on animals demonstrating an association between intranasal exposure to zinc salts and adverse influence upon olfactory function [74–94].

#### Human studies

In 1971, 301 workers at a zinc production plant were examined with regard to their olfactory and trigeminal function and compared with a control group comprising 63 workers at a machine factory [73]. The olfactory tests were performed by means of an olfactometer employing mint and dilute acetic acid (trigeminal stimulation). In comparison with the sensory threshold values of the control group the sensory thresholds of the exposed workers were statistically significantly elevated. The elevations were highest among the workers responsible for roasting the zinc ore. High concentrations – according to the author several times higher (without closer specification) than the limits in force at the time – of further strongly corrosive and irritant substances such as sulfur dioxide, sulfur anhydride, sulfuric acid, chlorine, hydrogen fluoride and others were however released in all three working areas (roasting, leaching, electrolysis) covered by the study, besides zinc oxide, zinc sulfate and metal dusts. Assessing the impact upon health to a particular substance is therefore difficult.

The link postulated by Seeber and Fikentscher between the effects of olfactory impairment observed among workers in a zinc chromate plant and exposure to chromium may perhaps equally be associated with zinc. This possibility was not examined in these studies [53, 54].

The suspicion that zinc in the form of a pharmaceutical component was capable of triggering olfactory dysfunction dates back to the 1930s. The suitability of a solution containing 1% zinc sulfate and 0.5% tetracaine (a topical anaesthetic) for use as a nasal spray for prevention of poliomyelitis infection was studied in Toronto in 1938 [28]. It was found not only that the desired protection was not achieved, but that some children and adults also developed anosmia. According to reports by the British Medical Journal and the Journal of Pediatrics, of 5233 children (for the most part aged between 3 and 10) treated in special clinics by otolaryngologists, approximately a quarter exhibited temporary anosmia [74, 75]. Unfortunately, the documentation contains no description of the diagnostic method, quantitative details of temporary and permanent olfactory dysfunction, or any indication whatsoever of systematic olfaction testing in the control group [74, 75]. Information on the control group and the test method were relevant insofar as recent studies indicate that olfaction is poorer in children than in adults: in the study by Sorokowska et al. of 1422 test subjects (aged between 4 and 80), children aged under 10 and adults aged over 70 performed worst in an identification test involving Sniffin’ Sticks with 16 different odours [95]. Tisdall et al. (1938) were in possession of data from a collective of an estimated 5000 further patients (children and adults) treated with zinc sulfate of which 44 were permanently anosmic as a consequence of the treatment. However, in this collective the 44 patients identified as having permanent olfactory dysfunction accounted for fewer than 1%, which is below the estimated figure for olfactory dysfunction in the wider population [16, 96].

Olfactory dysfunction occurring in patients following intranasal use of Zicam gel, claimed by the manufacturer to be “homeopathic” (according to Mossad, Zicam nasal gel contains 33 mmol/l zinc gluconate [97]) for prophylactic or therapeutic purposes against the symptoms of colds, was documented by Davidson and Smith, Alexander and Davidson, and Jafek et al. [76–78].

In 2009, the US Food and Drug Administration (FDA) issued warnings to consumers against three intranasal “Zicam” products containing zinc owing to the suspicion that they “may cause a loss of sense of smell”, possibly permanent [98]. The products concerned were then taken off the market in 2009.

The possible olfactory effect of a nasal insulin spray containing zinc as an additive was also the subject of recent discussion [99, 100]. Indeed, it is unclear how much of the zinc spray actually reaches the olfactory cleft. Notable in this context are the results of experiments by Herranz Gonzalez-Botas and Padin Seara, who examined the efficacy of the nasal gel form of application and ascertained that pigmented nasal gel is not detectable in the olfactory cleft following self-application by 16 test subjects [101].

#### Animal experimental and in vitro studies

The cytotoxic effect of Zicam was also demonstrated in vivo on mice and in vitro on human nasal tissue. Following instillation of Zicam by injection in the nasal cavities (15 μl per cavity), the olfactory epithelium was especially affected. The results of the behavioural test showed that treatment of the mice with Zicam led to olfactory dysfunction that still persisted two months after treatment. In the human nasal tissue samples, necrosis of the epithelial and subepithelial structures was observed following the application of Zicam [80].

Numerous further histological studies have been performed that illustrate the degenerative effect of zinc salts upon the olfactory epithelium in mice and fish [81–86]. Several studies in which ZnSO_4_ is used for experimental induction of anosmia in laboratory animals demonstrate that direct treatment of the olfactory mucous membrane with zinc sulfate solution impairs olfaction in mice, rats, hamsters and pigeons [81, 84, 87–91]. McBride et al. (2003) provide an overview of 22 behavioural studies on mice in which olfactory dysfunction was induced by means of intranasal irrigation with ZnSO_4_ [81].

Following intranasal zinc gluconate instillation on mice (33 mM, 50–100 μl per nostril), Duncan-Lewis et al. (2011) were able to demonstrate, by means of a behavioural test, a significant reduction in olfaction compared to control mice treated only with phosphate-buffered saline (PBS) [92]. Significantly weaker olfaction was also exhibited by mice following treatment with copper gluconate. Irrigation with magnesium gluconate yielded no differences. The olfactory dysfunction was reversible, since no differences in olfactory behaviour were observed in a further behavioural test performed one month after treatment.

Electroolfactograms and patch-clamp tests on isolated rat olfactory epithelia showed that exposure to zinc metal nanoparticles in the picomolar range had a significantly reinforcing effect upon the reaction of the olfactory neurons following olfactory induction, whereas Zn^2+^ ions in the same concentration led to a reduced response to olfactory stimulation [93].

The toxic properties of zinc oxide nanoparticles in the olfactory system of rats are presented by Gao et al.: once-off instillation of a suspension of zinc oxide nanoparticles led to significant damage of the olfactory epithelium and to inflammation reactions [94]. In addition, the exposed rats exhibited a change in sniffing behaviour and appeared no longer able to distinguish between vanillin diluted with distilled water and distilled water alone. In an in-vitro assay on primary human olfactory cells, Osmond-McLeod et al. demonstrated that zinc oxide nanoparticles are able to induce cellular stress reactions, inflammation reactions and apoptosis, but do not activate DNA repair mechanisms [79]. They established that the cellular reactions to zinc oxide nanoparticles with a coated surface were weaker.

### “Pesticides”

#### Human studies

As already reported by Doty the neurological functions of workers employed in structural fumigation involving the pesticides of methyl bromide and sulfuryl fluoride were compared in 2015 in a cross-sectional study with those of control persons [102]. Significantly weaker olfactory function tested with UPSIT was observed among the workers subject to high sulfuryl fluoride exposure. A corresponding observation was not made for the workers with high exposure to methyl bromide. It should be noted here that with the exception of 11 individuals, the majority of workers were subject to coexposure with methyl bromide. Clear distinction between the effects of methyl bromide and those of sulfuryl fluoride is therefore difficult, not least since division of the workers by the criteria of “high exposure to sulfuryl fluoride” and “high exposure to methyl bromide” is based upon statements made by the workers themselves in a questionnaire. Methyl bromide is highly toxic and harmful to the central nervous system. In animal experiments it also causes damage to the olfactory epithelium. According to the authors, further pesticides (including chlorpyrifos, organophosphates, carbamates, pyrethrins, organochlorine pesticides) with which the test subjects had come into contact during work or leisure revealed no significant association with the results of the olfactory test and the memory test involving patterns. During work, the fumigators were also exposed to “small amounts” of chloropicrin, which was used as an irritant warning substance during fumigation with sulfuryl fluoride and methyl bromide (no concentration stated). Chloropicrin is a highly irritant gas [103].

In one recently published study an impaired olfactory function was demonstrated in Latino farmworkers exposed to pesticides [104]. 304 farmworkers exposed to pesticides were compared with 247 non-farmworkers. At significantly greater self-reported lifetime pesticide exposure, the farmworkers required significantly higher concentrations for odour detection; the odour identification did not differ between the groups (Sniffin’ Sticks). Unfortunately, it is not specified which types of pesticides the farmers worked with. In this context it is interesting that an impaired olfactory function could be an early symptom of Parkinson’s disease (PD), and that pesticides are also suspected of inducing symptoms of Parkinson’s. A strong association between farmers with a PD diagnosis and a reduced sense of smell is shown by Shrestha et al. 2017 [105].

#### Animal experimental studies

Whereas methyl bromide can strongly damage the olfactory epithelium in animal tests [106], the available literature provides no clear indication of the effect of sulfuryl fluoride upon olfaction. Inflammation of the nasal tissue was reported after high exposure to sulfuryl fluoride (inhalation of 300–600 ppm) in rats and rabbits [107, 108].

### Formaldehyde

Olfactory dysfunction in humans caused by formaldehyde was described relatively early. Spealman [109] provides an indirect indication in that he cites the medical department of an airline that had rejected the use of deodorants containing formaldehyde in airliners on the grounds that formaldehyde was known to impair olfaction. Lehnhardt and Rollin mention the strange case of a company emergency response officer who developed anosmia allegedly owing to compulsive sniffing of a therapeutic agent that released formaldehyde [110].

#### Human studies

Four human studies involving formaldehyde and its association with olfactory dysfunction were identified [111–114].

In an epidemiological cross-sectional study by Holmström et al. of workers at a factory producing formaldehyde and formaldehyde-based products and workers exposed to both formaldehyde and wood dust, significantly reduced olfactory function was measured (sensory threshold test employing pyridine) [111]. Hisamitsu et al. demonstrated a significantly diminished olfactory function in medical students who were exposed to formaldehyde vapours during an anatomy course in Japan. It must be emphasised that all affected individuals already had a preexisting history of allergic rhinitis. Olfactory function was fully restored after a year [112]. The publications of Holmström et al. and Hisanitsu et al. [111, 112] are mentioned by Doty 2015 [4] and will not be presented in greater detail here.

Kilburn et al. describe the result of a survey involving 76 female histology technicians (average age 40.8) who were exposed to formaldehyde (0.2–1.9 ppm) and to other substances such as xylene and toluene during their work with histological preparations [113]. 22 of these women were exposed to formaldehyde for 1 to 3 h, 47 for over 4 h. In a survey, 32% of the women in each subgroup stated that their olfactory function was diminished. Among a control group of 56 non-exposed women (average age 39.5), this was stated by only 5%.

A study of 75 male workers (average age: 38) with occupational exposure either to formaldehyde alone (0.1–1.1 mg/m^3^ with exposure peaks of up to 5 mg/m^3^) or to formaldehyde and wood dust (0.6–1.1 mg/m^3^) detected pathological changes of the nasal mucous membrane in 72 individuals [114]. No differences in the histological findings were observed between the workers exposed solely to formaldehyde and those who had also been exposed to wood dust.

Based upon the results of long-term toxicological inhalation studies on laboratory animals, an NOAEL for nasal damage of 1 ppm formaldehyde was determined in the literature [115]. In their cross-sectional study, Holmström and Wilhelmsson observed a significant worsening of the sensory threshold even at formaldehyde concentrations below 0.37 mg/m^3^ (= 0.3 ppm, and corresponding to the German OEL) [111]. Irreversible damage to the sensory tissue need not however be anticipated below the irritation threshold, upon which the OEL is based.

#### Animal experimental studies

Inhaled formaldehyde (3.2 ppm) and acrylic aldehyde (0.67 ppm) led to degeneration of the respiratory epithelium in rats, inhaled acetaldehyde (750 ppm) to degeneration of the olfactory epithelium. It was demonstrated on the same species of laboratory animal that the effects of a combined exposure to these aldehydes can increase exponentially [116].

10 rats were exposed for 4 h per day for 7 days to 12.5 mg/m^3^ formaldehyde by Li et al. [117]. Examination of the olfactory bulbs and hippocampi of the exposed rats revealed severe morphological damage compared to an untreated control group. Reduced production of glutamate, gamma-aminobutyric acid and nitrogen oxide synthase was also detected in this damaged tissue. Since the publication was in Chinese, only the English abstract could be evaluated here.

Diminished olfactory function was determined in rats that had been subject for 30 min twice a day for 14 days to inhalative exposure to a formaldehyde concentration of 13.5 ± 1.5 ppm [118].

### Acrylates

#### Human studies

Gobba and Doty have already described the study by Schwartz et al. in their reviews: hundreds of workers were studied in a cross-sectional analysis at a factory producing acrylates and methacrylates [119]. The workers were divided into four classes according to their exposure. No association was established in the first instance between exposure and the results of olfactory tests (UPSIT, *p* = 0.09). By means of an embedded case-control study, a dose-effect relationship was observed between olfactory dysfunction and cumulative exposure; the effect appeared to be reversible. The risk of developing olfactory dysfunction was greatest in the group of non-smokers.

Two studies were, however, not mentioned by Gobba or Doty:

In a cross-sectional study of 175 workers exposed to methyl methacrylate and a control group of 88 non-exposed workers employed in the same production unit for acrylic glass sheet casting, performance of the Rhino Identification Test (6 tested aromas, very similar in design to the UPSIT test) revealed only a single hyposmic case in the exposed group [120]. At 58.3%, the proportion of smokers was higher in the exposed group than in the control group (34.1%). Over an 8-h shift, exposure lay between 25 and 100 ml/m^3^ in 1988 and between 10 and 50 ml/m^3^ in the period from 1989 to 1994. The average exposure duration was 9.6 ±7.1 years. With the exception of 2 workers who were additionally exposed briefly to formaldehyde up to 1990 and 4 workers who additionally had contact with acrylonitrile, and a further 2 workers who were additionally exposed to both formaldehyde and acrylonitrile, all workers were exposed solely to methyl methacrylate. These results permit the conclusion that at exposures of up to 50 ml/m^3^ methyl methacrylate, olfactory function is not harmed.

In a more recent exposure study, 20 healthy male volunteers (non-smokers, aged 20–62) were exposed in an exposure chamber once to 49.2 (±1.4) ppm methyl methacrylate for 4 h. Following exposure, no changes occurred in the olfactory threshold for n-butanol, which was measured by means of Sniffin’ Sticks, nor had the measured mucociliary transit time (time from introduction of a saccharin particle in the lower nasal vestibule to perception of a sweet taste in the throat) changed. Also unchanged were the measured concentrations of protein and mRNA markers of inflammation in the nasal secretion and respiratory epithelium. Furthermore, only minor differences were observed in the mental state, which was evaluated by questionnaire. The authors concluded from this that acute exposure to 50 ppm methyl methacrylate causes no inflammatory changes to the respiratory nasal mucous membrane, and that in view of the absence of a rise in the olfactory threshold following exposure to 50 ppm methyl methacrylate, this dose is not sufficient to cause toxic damage to the olfactory epithelium. These results of acute exposure cannot be readily transferred to chronic conditions [121].

#### Animal experimental studies

Chronic exposure to 100 ppm methyl methacrylate can cause degeneration and atrophy of the olfactory epithelium in rats. It must be considered that the activity of the carboxylesterase in the olfactory epithelium of the nasal mucous membrane is considerably higher in rats than in humans. The unspecific carboxylesterase hydrolyses methyl methacrylate to methyl acrylic acid, which is responsible for the local toxicity [122].

### Styrene

#### Human studies

Three studies have already been listed by Doty [4]:

Cheng et al. [123] compared injection moulding workers exposed to styrene with non-exposed workers. At the end of a working day, a slight but significant reduction in olfactory function was detected in the olfactory threshold test employing 1-butanol, whereas the initial situation at the beginning of the shift had been the same. An identification test employing 7 odours (Connecticut Chemosensory Clinical Research Center (CCCRC) olfactory test) revealed no differences before and after the shift. According to Doty [4] these results support the “concept that the olfactory thresholds reflect adaptation rather than sustained neurological damage”. Unfortunately, no exposure measurements were performed in this study.

Other epidemiological studies of workers in the glass fibre reinforced plastic industry revealed no relationship between styrene exposure and a general deterioration in olfactory function [124, 125]. In both studies the olfactory threshold for styrene was significantly higher among the exposed workers. According to Dalton et al., this is also explained by an adaptation effect, which leads to a reversible reduction in sensitivity. This has already been frequently observed for volatile substances in industry or the laboratory and is correspondingly well documented [126]. Whereas the identification test revealed no differences between exposed and non-exposed individuals in the study published in 2003, the identification test published in 2007 resulted in a significant difference between the workers with high vs. low exposure [125]. Dalton et al. state that the proportion of individuals showing poor values in the identification test is substantially higher in both groups (40 and 20%) than in the normal population (10%) [125]. Even with the aid of multiple regression analysis, no association was demonstrated between the results of the identification test and the exposure values measured at present or in the past in the group of workers subject to high exposure. The authors therefore suspect that the high proportion of immigrants in the exposed group had influenced the results of the test, and they do not consider the results to be valid evidence of impairment of human olfactory function by workplace exposure to styrene.

#### Animal experimental studies

In two studies on rodents conducted in 1997 and 1998, already cited by Doty 2015, styrene exposure of between 20 and 50 ppm led to lesions of the olfactory epithelium [127, 128]. Green et al. assumed that the nasal lesions induced in mice by exposure to styrene were caused by styrene oxide, which cannot be detected in the human nasal epithelium [129].

### Organic solvents and mineral oil products

#### Human studies

The publications by Schwartz et al. [130], Sandmark et al. [131] and Ahlstrom [132] are epidemiological studies evaluating a possible association between solvent exposure and impairment of olfaction. They are also cited by Gobba and Doty:

With consideration for smoker status, the UPSIT identification test performed by Schwartz et al. [130] yielded a significant dose-dependent deterioration in olfactory function of workers exposed to solvent in paint manufacture with rising lifetime exposure among workers who had never smoked. This effect was not observed among workers who had always smoked. The results remained the same when the confounders of age and cultural background (vocabulary testing) were taken into account. Schwartz et al. suspect that the induction of cytochrome P450 enzymes by cigarette smoke leads to an increase in the metabolism and thereby to detoxification of organic olfactotoxins before they reach the olfactory epithelium. The exposed workers who had always smoked performed worse in the UPSIT olfactory test compared to reference values, albeit not with dose dependency. The best olfactory function was exhibited by the workers with the lowest exposure who had never smoked.

In an UPSIT identification test on painters, no impairment of olfactory function was observed following multiple regression analysis including consideration for age and smoker status [131]. Quantitative exposure measurement was not performed; the exposure was described as low to moderate. Tank cleaners studied by Ahlstrom et al. exposed to mineral oil products exhibited elevated olfactory threshold values for heated oil vapour and n-butanol; these were still within the normal range for n-butanol. Oil vapour had not been studied in this respect before. To determine whether a relationship existed between the elevated threshold values and the interval between the most recent exposure and the olfactory test, the exposed workers were assigned to three groups with intervals of different duration (1 day to over 30 days) between exposure and the olfactory test. It was found that the tank cleaners who had been exposed just one day prior to the test exhibited higher threshold values for all tested substances than those who had not. A further test of the subjectively perceived odour intensity of all 4 substances tested (pyridine, dimethyl disulfide, n-butanol and heated oil vapour) revealed that at the lowest stimulus concentrations, the exposed persons exhibited lower odour intensity values than the control subjects [132].

Muttray et al. refer to one Russian and two Polish studies of the olfactotoxic effect of adhesives containing petroleum: of workers at a Russian shoe factory who had been exposed to an adhesive containing a petroleum mixture (“Galoscha”), ethyl acetate and butyl acetate (solvent concentration: 220–300 mg/m^3^), over 25% suffered atrophy of the nasal mucous membrane owing to inflammatory processes, 31% suffered the loss of olfactory function, and among the remainder, olfactory function was impaired in comparison to a control group [133]. Among 205 workers who used a rubber adhesive containing petroleum and were exposed to it up to a level of approximately 3000 mg/m^3^, olfactory dysfunction as a result of rhinitis arose after only a few years. Muttray also cited a study of 547 petroleum chemical workers. According to the abstract of this publication (article in Polish, [134]) the senses of smell and taste were examined. Hyposmia was found in 238 (43.5%) and anosmia in 50 (9.2%) subjects. Dysgeusia (distortion of the sense of taste) was identified in 319 (58.3%) subjects, with no reaction at all in 102 (18.6%) persons. In the control group (100 cotton industry workers) dysgeusia was determined in 69%, anosmia in 24% of subjects. According to Muttray the exposure was described insufficiently.

#### Animal experimental studies

In an experiment conducted by the US National Toxicology Program (NTP), no conspicuous lesions of the olfactory epithelium were observed in rodents following inhalation of tetrahydrofurane [135]. In a further NTP study, degeneration of the olfactory epithelium was observed in all exposed mice following exposure to cyclohexanone in drinking water [136].

The capacity of 2-ethylhexanol to impair both the olfactory epithelium and the olfactory bulb in mice is demonstrated by Miyake et al. [137]: subchronic inhalation of 2-ethylhexanol induced degeneration of the olfactory epithelium at a lowest observed adverse effect level of 20 ppm. Following exposure for three months, changes in the olfactory bulb (such as a reduction in the glomeruli diameter, increase in the microglia) were also observed.

## Mechanism of olfactory dysfunction

Odorants enter the nose orthonasally or retronasally and bind to receptors on the cilia of the olfactory sensory neurons in the olfactory epithelium. The stimulus is converted here into signals and forwarded to the brain. Whereas the respiratory epithelium lines the greater part of the respiratory tract, possesses no sensory neurons, and as the epithelium of the respiratory tract has the functions of keeping the tract clean and of moistening and warming the breathing air, the human olfactory epithelium is limited to a small area of approximately 2 × 5 square centimetres in the superior turbinate bone [138, 139]. Besides the approximately 10–30 million olfactory (bipolar) neurons, the olfactory epithelium consists of basal cells and supporting cells. The functions of the supporting cells (sustentacular cells) include biotransformation of xenobiotics [140].

Olfactory dysfunction can be caused by impairment of transport of the odorant molecules to the olfactory epithelium (conductive cause) and/or by sensorineural dysfunction.

Exclusively conductive or sensorineural dysfunction are rare; a combination of both is common. A possible conductive cause is for the transport of odours to the olfactory epithelium to be obstructed: in sufficient doses and with sufficiently long exposure, allergens and substances irritative and corrosive to the mucous membrane may cause rhinitis accompanied by inflammatory swelling of the mucous membrane. By obstructing nasal breathing, this can lead to anosmia, which is reversible once inflammation has subsided [141]. Conductive olfactory dysfunction may also be caused by chronic irritation by dusts such as calcium carbonate or cement [110].

Sensorineural causes can be broken down further according to the affected olfactory structure into damage to the olfactory epithelium, damage to the olfactory fibres, and central nervous effects.

The neurons of the olfactory epithelium are directly exposed to environmental influences. Their apical end is in direct contact with the external environment and not separated from it by synapses, and at their basal end they have direct access to the brain via their nerve fibre (axon): inhaled hazardous substances may therefore lead to impairment of the olfactory epithelium and, owing to the intracranial connection, consequently to damage to the bulbus olfactorius and higher brain regions [142].

Compared to all other neurons, those of the olfactory epithelium in the nose of vertebrates are unique in a further respect: they are able to self-renew after a certain lifespan (approximately 30–60 days in the case of rodents; data for human beings are not available [140]) by the differentiation of basal cells. The capacity for regeneration probably decreases with increasing age.

Of the industrial chemicals discussed in the present article, exposure to cadmium, nickel, zinc, cyclohexanone, styrene, acrylates and formaldehyde resulted in damage to the olfactory epithelium in animal experiments. Acute high exposure to reactive and soluble gas such as formaldehyde, chlorine and ammonia may directly damage the respiratory and sensory epithelium in the nose and induce lesions up to and including necroses, resulting in olfactory dysfunction [143]. In animal experiments, it was observed histologically that the olfactory epithelium appears “naked” following toxic exposure. It is described in these terms in one textbook of olfactory dysfunction and dysgeusia [142]. The textbook documents the histological finding of an olfactory biopsy of an anosmic patient following exposure to chlorine gas, by way of a photograph on which the loss of the olfactory receptor neurons is clearly visible.

The spectrum of histological changes that can occur following chronic exposure to irritants encompasses inflammation reactions, degeneration, atrophy, necrosis, keratinisation, hyperplasia, metaplasia and neoplasia [22]. Specific observations include both regeneration of the damaged epithelium by basal cells and metaplastic processes, besides a reduction in the number of olfactory cilia, degeneration of sensory and sustentacular cells and of the Bowman’s glands and nerve fascicles, and necrosis of individual cells. The olfactory epithelium can therefore be replaced by respiratory/squamous epithelium.

Unspecific necrosis of cells of the olfactory epithelium can be caused by reactive irritative substances such as chlorine and sulfur dioxide, whereas cell-specific toxicity, affecting for example the sustentacular cells or sensory cells, can be induced by dibasic esters and methyl bromide. Methyl methacrylate is known to be metabolised by a non-specific esterase in the olfactory mucus to methanol and to methacrylic acid, which destroys the receptor cells [120]. The effect may be reversible when the basal cells, from which new receptor cells can form, remain intact.

In the olfactory epithelium itself, enzymes, particularly of the cytochrome P450 family (CYP), are expressed which metabolise toxic substances and can therefore both detoxify and toxify. The sustentacular cells express CYP proteins, which are responsible for the oxidation of xenobiotics. If these cells are damaged or destroyed, the metabolic activity of the CYP enzymes is also inhibited. Further factors, such as genetic polymorphisms, tobacco smoke and other exogenous substances (diallyl sulfide, m-xylene, 8-methoxypsoralen) may also have an inhibiting effect upon the respiratory CYP isoenzymes [22].

The activity of these enzymes decisively influences the toxic effects of many inhaled substances upon the respiratory and olfactory mucous membrane. Whereas, following inhalation, many toxic substances can be converted by metabolisation with the aid of these enzymes to intermediate products of lower toxicity, xenobiotic substances also exist that do not become toxic reactive intermediates until after metabolisation, as in the example of metabolic activation of carcinogenic nitrosamines from tobacco smoke catalysed by CYP2A13. CYP2A13 is strongly expressed in the nasal mucous membrane and is able to catalyse a range of atmospheric hazardous substances such as naphthalene, styrene and toluene [22].

The induction by cigarette smoke of CYP proteins (including CYP1A1) in the olfactory mucous membrane has been demonstrated in rats [144, 145]. Metabolisation to more toxic vs. less toxic products or intermediates may also potentially explain the apparently partly contradictory results regarding the impairment of olfactory function by inhaled substances in smokers. In an exposure study involving acrylates and methacrylates for example, the risk of developing olfactory dysfunction was greater in the non-smoker group [119]. Since tobacco smoke damages the sustentacular cells and thus inhibits enzymatic biotransformation, the creation of toxic intermediary products can also be prevented as a result, and the nasal function thus protected. Conversely, it is also conceivable that inhibition of the enzymes reduces detoxification of the substances, resulting in greater damage to the olfactory mucous membrane.

Altogether, little is known of central nervous effects leading to toxic olfactory dysfunction by substances. Following binding of the odorant molecules to the olfactory receptors, the stimulus information is further transmitted along the bundled axons of the olfactory receptor neurons (nervus olfactorius) through holes in the cribriform plate (lamina cribrosa) into the brain to specific glomeruli in the bulbus olfactorius. The glomeruli consist of the projections of the receptor neurons, which form synapses with the dendrites of pyramid-like mitral cells and of interneurons. Over 1000 axons of olfactory receptor neurons with the same receptor type project onto the dendrites of a single mitral cell [138]. Cadmium and manganese can be detected in the bulbus olfactorius of animals (see above, [38, 39, 63, 68, 72]) and in humans: Baader (1952) was able to determine atrophy of the nasal mucous membrane and an intensive yellow colouring of the olfactory bulb following the autopsy of an affected worker exposed to cadmium, which led him to suspect that cadmium reached the brain via the olfactory pathway [146]. After MRT research on non-human primates exposed to aerosolised manganese sulfate (particle diameter: 1.04–2.12 μm) had already demonstrated transport of manganese via the olfactory pathway and accumulation of manganese in the olfactory bulb [147], increased deposition of manganese in the olfactory bulb was also detected on 7 welders by means of functional magnetic resonance tomography [63].

Over a decade ago, Oberdörster et al. voiced their suspicion that inhaled nanoparticles were able to penetrate the brain through the olfactory nerve [148]. It was shown recently that following inhalative exposure on mice, “quantum dots” (cadmium selenide and zinc sulfate nanocrystals) enter the brain and olfactory bulb by direct axonal transport from the nose; activation of microglia in the olfactory bulb was observed at the same time [149]. In humans, the progress of direct transfer of a substance – in this instance following application of thallium-201 (^201^TI) – from the nose, via the olfactory epithelium and the olfactory nerve, into the brain was first demonstrated in 2010 by a Japanese team with the aid of SPECT computer tomography and magnetic resonance tomography [150]. Interestingly, this method was also used recently to document that significantly lower migration of ^201^TI into the olfactory bulb occurred in patients with an olfactory dysfunction [151]. Neurodegeneration was also observed following exposure to manganese: intranasal exposure of mice to manganese chloride in saline solution led to increased necrosis of the granule cells in the bulbus olfactorius. Generally acting as inhibitory interneurons, the granule cells contribute to lateral inhibition and thus to contrast intensification of the activity patterns, sharper discrimination between different odours [138, 152].

At molecular level, a toxin-mediated reduction in the release of neurotransmitters could contribute to olfactory dysfunction: following intranasal application of a dilute cadmium chloride solution in mice, Czarnecki et al. demonstrated anosmia by means of a behavioural test, and a dose-dependent reduction in the odour-induced release by the olfactory nerve of neurotransmitters into the olfactory bulb [40]. Regarding the possible mechanism of action, Czarnecki et al. speculate that as a divalent cation, Cd^2+^ disrupts calcium-mediated neuronal signal transduction by blockage of the transmembrane calcium channels. Earlier studies had shown that release of neurotransmitters from the presynaptic terminals of the olfactory receptor neurons requires an influx of calcium ions via N-type calcium channels. This permits the presumption that owing to the mechanism of amplifying signal transduction, even a minor cadmium-mediated reduction in the presynaptic calcium influx may have a major influence upon the entire release of transmitters by the olfactory nerve.

Diminished release of neurotransmitters following exposure to manganese was also demonstrated by Moberly et al. and Guilarte et al. in the olfactory bulb and in the basal ganglia [153, 154]. Whereas no histopathological changes were evident, the study by Moberly et al. showed that intranasal instillation of as little as 2 μg MnCl_2_ in mice was sufficient to cause significant changes in odour-induced neurotransmitter release from the olfactory nerve [153]. At 200 μg, the reduction was 90%. This effect may be presumed attributable to a central nervous effect of the manganese, since the release of neurotransmitters was reduced not only after odour stimulation, but also after electrical stimulation.

## Evaluation

As yet, few reasonably reliable epidemiological studies have been produced of the olfactotoxic effect of industrial chemicals. Interpretation of these publications revealed that cadmium, nickel, chromium and formaldehyde encountered under workplace conditions can be assumed with reasonable certainty to impair the human olfactory function. This finding is supported for the most part by the results of animal experiments.

The level of evidence for an empirically demonstrated relationship between the development of olfactory dysfunction and exposure to potentially olfactotoxic substances at workplaces can be considered comparatively weak in the majority of cases for the following reasons:

The majority of epidemiological studies of this specific occupational problem are cross-sectional involving heterogeneous collectives in which the individual levels and durations of exposure often differ widely. Despite high variance in olfactory function within the wider population, the studies discussed in this article consider comparatively small groups of people. Aside from the large-scale poliomyelitis prevention campaign employing zinc sulfate nasal spray, only Pyatayev [73] and Casjens et al. [67] have studied several hundred individuals. According to Mücke and Lemmen [126], olfactory sensitivity in a population approximately follows a normal distribution: “(…) although the breadth of perceived concentrations may differ by a factor of up to a hundred within the group (…)”. To our knowledge, no valid specific prospective cohort studies of work-related olfactory dysfunction other than Casjens et al. are available. However cross sectional analysis of data collected in large nationwide surveys such as the US National Health and Nutrition Examination Survey (NHANES) provides estimates representative at national level of the prevalence of taste and smell impairment in the US population and also identifies exposure variables correlating with olfactory dysfunction [155–158]. In the German National Cohort (GNC), a random sample of the general population will be taken by 18 German regional study centres, including a total of 100,000 women and 100,000 men aged 20–69 years, with the aim of increasing understanding of the role of particular risk factors in the development of major forms of chronic disease. Special emphasis will be placed on selected lifestyle factors, but also on occupational and environmental influences. One functional measurement that will be performed is of olfactory function [159].

Details of the reversibility or persistence of olfactory dysfunction induced by working substances are often unavailable. More follow-up studies of workers who have changed jobs could be useful. Olfactory dysfunction is not easily quantified. Standardised olfactory tests were not developed until recent decades. Comparison between the study results is rendered more difficult by differences between the test methods employed. Olfactory self-assessment tends to be unreliable and it has been shown that people do not perform well when compared with psychophysical testing [6].

Monitoring of all relevant confounders is difficult in workplace assessments. Olfactory function is known to decrease with increasing age. Indications exist of differences between the sexes, and differences in ethnic background may also be associated with differences in olfactory function. Olfactory function may be affected by alcoholism and smoking behaviour, medication, hormonal impacts, hunger and environmental influences [160–162].

The problem of mixed exposure exists at many workplaces, as described for example in Chapter 2 with regard to the metals industry. Firefighters, paramedics and police officers may be exposed to a range of different dusts and fumes in disaster situations. An impressive example is provided by Altman et al. and Dalton [24, 163]: following the terror attack on the World Trade Center in New York in 2001, many responders and local residents exhibited impairment of olfactory function, in addition to the symptoms of other conditions. Workers are also exposed in day-to-day operations in sewage works and landfills to a range of irritants and agents corrosive to the mucous membrane which may lead to impairment of the sense of smell [164].

Comparison of the concentrations in the studies cited with the current situation at workplaces in central Europe is hampered by the gaps in the information provided by the studies on the sampling and analysis methods. Convincing evidence that the olfactory dysfunction triggered by olfactotoxic industrial chemicals is the critical, namely most sensitive toxicological effect of these substances, has not yet been provided.

In general, relevant physiological differences, and also anatomical differences, must be considered when the results of animal experiments are transferred to humans. Whereas humans are oronasal breathers, rodents are obligate nasal breathers. In humans, the olfactory epithelium covers approximately 3% of the nasal cavity; in rats for example, it covers as much as 50%. The situation is similar for mice, rabbits and dogs. Owing to the olfactory epithelium’s sheltered location in human beings, less than 15% of the air inhaled through the nose reaches it – substantially less than for example in rodents [22].

Certain differences are observed in the activity of metabolic enzymes in laboratory animals and humans. High concentrations of the two cytochrome P450 isoforms which are required for the conversion of styrene to styrene oxide were for example detected in respiratory fractions of rats and mice, but not in those of humans [165]. At styrene exposure concentrations resembling those of occupational exposure, mice and rats exhibit extensive damage, whilst humans exhibit no indication of a dose-dependent long-term change in olfactory function [124, 125, 127, 128].

## Conclusion and future prospects

An impaired olfactory function not only diminishes the quality of life of those affected, but can also present a workplace hazard for the workers and the company if it results in hazardous substances no longer being detected sufficiently quickly.

Isolated indications that chronic occupational exposure to comparatively low levels of the substances stated above has a harmful impact upon olfactory function should be examined more closely, as should the issue of the effects’ reversibility. In this context, the significance of neuroplasticity must be considered, which seeks to compensate for failures in the sensory apparatus.

Since many affected individuals are shown not to be aware of their olfactory dysfunction, greater consideration should be given to regular check-ups of the olfactory function of workers at certain workplaces. At present, olfactory check-ups are performed only in very few working areas. In Germany, tasks associated with room fumigation and room disinfection are an example [3, 166]. SUVA, the Swiss accident insurance institution, recommends olfactory tests as part of occupational health monitoring for persons working with smellable toxic and explosive hazardous substances [167]. The substances occurring at the workplace concerned should be included in the olfactory test, in order for selective anosmia for certain industrial chemicals also to be detected [126]. Normal accustomisation effects such as adaptation and habituation that may impair the sensing of possible hazardous substances at the workplace should be considered at the same time.

An olfactory test not only ensures that the employees with potential contact with smellable hazardous substances are able to sense them and their warning function, but could also serve as a means of early detection of pathological changes. Rose et al. and Mascagni et al. for example discuss the possible use of olfactory dysfunction as an early indicator of toxic effects induced by cadmium [34, 37]. It is also possible that other neurotoxic substances not only cause direct damage to the olfactory epithelium, but also damage the central nervous system following transport through the nose into the brain. Accordingly, olfactory dysfunction could have an early-warning function for potential damage to the brain induced by the same neurotoxins. Olfactory dysfunction also serves as a marker for certain neurodegenerative diseases. In conjunction with the idiopathy of Parkinson’s syndrome, olfactory dysfunction may arise as early as four to six years before the first motor dysfunction typical of Parkinson’s, such as shaking and muscular rigidity [16, 168].

Not only must employees with severe olfactory dysfunction be advised that they are able to detect smellable hazardous substances only poorly, if at all, suitable measures must also be taken at work and in private life to prevent a hazard arising.
